# Self-Assembling Polypeptide Hydrogels as a Platform to Recapitulate the Tumor Microenvironment

**DOI:** 10.3390/cancers13133286

**Published:** 2021-06-30

**Authors:** Dariusz Lachowski, Carlos Matellan, Ernesto Cortes, Alberto Saiani, Aline F. Miller, Armando E. del Río Hernández

**Affiliations:** 1 Cellular and Molecular Biomechanics Laboratory, Department of Bioengineering, Faculty of Engineering, South Kensington Campus, Imperial College London, London SW7 2AZ, UK; d.lachowski15@imperial.ac.uk (D.L.); c.matellan16@imperial.ac.uk (C.M.); j.e.corteslopez@imperial.ac.uk (E.C.); 2 Department of Materials, Manchester Institute of Biotechnology, Faculty of Science and Engineering, The University of Manchester, Oxford Road, Manchester M13 9PL, UK; a.saiani@manchester.ac.uk; 3 Department of Chemical Engineering and Analytical Science, Manchester Institute of Biotechnology, Faculty of Science and Engineering, The University of Manchester, Oxford Road, Manchester M13 9PL, UK; 4 Manchester BIOGEL, Mereside, Alderley Park, Alderley Edge, Cheshire SK10 4TG, UK

**Keywords:** tumor acidosis, pH, tumor microenvironment, self-assembling polypeptides, cell culture

## Abstract

**Simple Summary:**

The tumor microenvironment is characterized by increased tissue stiffness, low (acidic) pH, and elevated temperature, all of which contribute to the development of cancer. Improving our in vitro models of cancer, therefore, requires the development of cell culture platforms that can mimic these microenvironmental properties. Here, we study a new biomaterial composed of short amino acid chains that self-assemble into a fibrous hydrogel network. This material enables simultaneous and independent tuning of substrate rigidity, extracellular pH, and temperature, allowing us to mimic both healthy tissues and the tumor microenvironment. We used this platform to study the effect of these conditions on pancreatic cancer cells and found that high substrate rigidity and low pH promote proliferation and survival of cancer cells and activate important signaling pathways associated with cancer progression.

**Abstract:**

The tumor microenvironment plays a critical role in modulating cancer cell migration, metabolism, and malignancy, thus, highlighting the need to develop in vitro culture systems that can recapitulate its abnormal properties. While a variety of stiffness-tunable biomaterials, reviewed here, have been developed to mimic the rigidity of the tumor extracellular matrix, culture systems that can recapitulate the broader extracellular context of the tumor microenvironment (including pH and temperature) remain comparably unexplored, partially due to the difficulty in independently tuning these parameters. Here, we investigate a self-assembled polypeptide network hydrogel as a cell culture platform and demonstrate that the culture parameters, including the substrate stiffness, extracellular pH and temperature, can be independently controlled. We then use this biomaterial as a cell culture substrate to assess the effect of stiffness, pH and temperature on Suit2 cells, a pancreatic cancer cell line, and demonstrate that these microenvironmental factors can regulate two critical transcription factors in cancer: yes-associated protein 1 (YAP) and hypoxia inducible factor (HIF-1A).

## 1. Introduction

The tumor microenvironment (TME) is a complex milieu of cancer-associated cells and molecules, whose chemical and biophysical properties modulate cancer cell behavior, promote cancer progression, and facilitate metastasis. The critical role of the tumor microenvironment in cancer has prompted the development of new research platforms that can mimic its microenvironmental properties. These new platforms include more advanced 2D cultures that recapitulate the mechanical properties of the tumor extracellular matrix (ECM) [1], co-culture models that incorporate cancer associated fibroblasts (CAFs) [2,3] or immune cells [4], constructs that mimic the three-dimensional architecture of tumors (organotypic and bioprinting) [5,6], tumor organoids and spheroids [7,8] and microfluidic approaches (tumor-on-a-chip) [9,10]. The use of these novel technologies has revealed fundamental insights into the role of the tumor microenvironment as a driver of cancer, thus, highlighting the need to understand and recapitulate the complex extracellular context of tumors.

Two key microenvironmental hallmarks characterize the tumor microenvironment: the increased stiffness of the aberrant ECM (tumor desmoplasia) and the low extracellular pH of the interstitial fluid (tumor acidosis). CAFs, activated by their crosstalk with cancer cells, remodel the native ECM through the deposition of ECM fibers, the secretion of matrix metalloproteinases (MMPs) and physically through their contractility [11,12]. This matrix remodeling results in a high substrate stiffness, which, in turn, supports the activation of CAFs, guides the migration of cancer cells and promotes epithelial-to-mesenchymal transition (EMT) [13,14,15]. Conversely, tumor acidosis is the result of the metabolic reprogramming of cancer cells from oxidative phosphorylation to glycolytic metabolism, which generates lactic acid as a byproduct [16,17]. 

To maintain a stable intracellular pH, cells extrude the excess protons generated in this process through a variety of proton pumps and voltage-gated proton channels [18], which, in turn, accumulate protons in the interstitial fluid, thus, resulting in an acidic microenvironment. Tumor acidosis promotes cancer cell migration, metastasis and immune cell infiltration and contributes to the inhibition of immune surveillance and the development of chemoresistance [19,20]. Interestingly, this switch toward glycolysis occurs even in high oxygen availability (normoxia), a phenomenon known as the Warburg effect [20]. 

Significant research has focused on the development of biomaterials that can serve as cell culture substrates with tunable stiffness (Table 1) [1]. Natural (collagen and alginate), synthetic (polyacrylamide and polyethylene glycol) and hybrid (methacrylated hyaluronic acid) hydrogels have been used as 2D or 3D substrates [21] to investigate stiffness-driven phenotypic changes [22], invasion [23] and chemoresistance [24,25] in cancer cells. However, the simultaneous mimicking of both microenvironmental factors remains unexplored. Understanding how the two factors contribute to modulating cancer cell behavior, as well as the interplay between them, requires the use of culture platforms that allow for the independent tuning of both the pH and substrate stiffness. 

This kind of system, however, has remained elusive. Collagen gels typically require neutral or alkaline pH for gelation [45,46] (although fibrils can form at a lower pH [47]), and their fiber architecture and mechanical properties widely depend on pH [46,48]. Some polymeric hydrogels used as cell culture matrices are pH-sensitive alone or in composites, including chitosan and polyacrylic acid [49,50,51], while polyacrylamide (PAA), the standard substrate for mechanobiology studies, is ill-suited for 3D cultures due to the cytotoxicity of its components [21]. 

Here, we evaluate a new biomaterial based on a tunable self-assembled polypeptide network (PeptiGel) and demonstrate that its mechanical properties remain stable over a range of pH values (6.0–7.4) and temperatures (37–40°C), thereby, enabling independent tuning of pH, temperature and stiffness. We then assess these matrices as cell culture platforms to recapitulate the tumor microenvironment and demonstrate that both substrate stiffness and extracellular pH positively regulate the proliferation and survival of pancreatic cancer cells, while temperature increases apoptosis. Finally, we demonstrate that pH and stiffness modulate the expression of two master regulators in cancer, yes-associated protein (YAP) and hypoxia inducible factor 1 (HIF-1A), suggesting an interplay between cancer metabolism and mechanotransduction. 

## 2. Comprehensive Review

### 2.1. Collagen

Collagen is one of the most abundant and ubiquitous fibrous proteins in the human body and one of the main components of the extracellular matrix (ECM). There are 29 different collagen types that have been identified, of which collagen type I is the most abundant and frequently used biomaterial. Collagen fibers present a hierarchical fibrillar structure over several scales [52] that can self-assemble into hydrogels through physical crosslinking. The hydrogel architecture and fibril self-assembly depends on multiple fabrication parameters, such as temperature and pH [48], notably requiring a neutral or alkaline pH to form stable hydrogels [45,46]. Collagen is among the most used biomaterials owing to its physiological nature and ability to mimic the native ECM. It excels in 3D culture applications to observe matrix remodeling, degradation or cell migration. 

The mechanical properties of collagen gels can be controlled with the tuning fabrication parameters, primarily the collagen concentration, although the range of stiffness that can be achieved without additional chemical crosslinking is limited (<1 kPa) [26,27]. Temperature and pH also affect fibril self-assembly, which ultimately impacts the gel architecture, porosity and, thus, mechanical properties [48]. 

Different methods of crosslinking have been explored to improve the mechanical properties of collagen hydrogels in order to mimic tissues of higher stiffness, including chemical crosslinkers, such as glutaraldehyde [53], carbodiimides [28,29,54] or genipin [55], as well as physical crosslinkers in the form of UV light or dehydrothermal treatment (DHT) [44,52]. Composites of collagen with other materials can also be used to combine its biological properties with the mechanical robustness of synthetic materials [54,55,56]. However, as a natural material, collagen suffers from batch-to-batch variability, limiting the standardization and reproducibility of mechanical properties [21].

Due to its physiological nature, collagen natively presents cell adhesion motifs, and cells readily attach via integrins and spread on collagen substrates without requiring additional functionalization [57]. While this biological functionality can be highly desirable in many applications, such as tissue engineering, it can be detrimental in mechanobiology studies, since the ligand presentation and density cannot be decoupled from the mechanical properties. Collagen hydrogels also undergo significant matrix contraction and can be remodeled by encapsulated cells [58,59], resulting in changes to the architecture and mechanical properties. 

### 2.2. Alginate

Alginates are a family of natural polysaccharides composed of β-d-mannuronic acid (M) and α-l-guluronic acid (G) arranged in the form of block co-polymer subunits [60]. Alginate is derived from brown algae, offering different formulations with a wide range of molecular weights and excellent biocompatibility. The formation of hydrogel networks is achieved via ionic crosslinking with multivalent cations, typically Ca^2+^, although covalent crosslinking is also possible [61]. RGD peptides or other cell adhesion ligands are usually added to alginate to improve cell adhesion [62]. 

The mechanical properties of alginate hydrogels can be tuned through a variety of parameters, including the concentration and molecular weight (MW) of alginate. Increasing the concentration or the molecular weight of the polymer results in hydrogels with higher stiffness due to an increase in polymer chain entanglement and long-range interactions between polymer chains [31,61]. However, high MW results in high viscosity formulations that are difficult to work with, especially in bioprinting applications. Formulations that rely on a combination of high and low MW alginate are preferred, as they offer low viscosity while retaining good mechanical properties [60,63]. 

The choice of crosslinker and the ratio of alginate to crosslinker are also key parameters to control the hydrogel stiffness. While calcium chloride (CaCl_2_) is the most common choice of ionic crosslinker, calcium sulphate (CaSO_4_) and calcium carbonate (CaCO_3_) have also been used, resulting in hydrogels with different mechanical properties due to changes in the gelation rate [64]. Generally, slower gelation results in more uniform gels with higher mechanical strength and rigidity [31,64]. 

The choice of medium (e.g., PBS vs. DMEM) can likewise affect the mechanical properties of alginate due to differences in the solubility of ionic crosslinkers in different media [61,64], which impact the gelation time. Similarly, increasing the crosslinker to alginate ratio increases the Young’s modulus of the resulting hydrogel due to an increase in the crosslinking density [31,64]. The potential for covalent crosslinking through poly(ethylene glycol) diamines [65], adipic acid dihydrazide [66] and 2-aminoethyl methacrylate [67] offers another dimension to regulate the mechanical properties of alginate gels, either alone or in combination with ionic crosslinking. 

Alginate gels have been used as 2D substrates to study the effect of rigidity and adhesion ligands on the morphology and cell/substrate interaction in chondrocytes [62] and to study the effect of stress relaxation on cell behavior [68]. However, much mechanobiology research with alginate gels has focused on 3D due to its excellent suitability for bioprinting. Three-dimensional alginate matrices of different rigidity have been used to investigate the effect of substrate stiffness on the differentiation of mesenchymal stem cells (MSCs) in 3D, and to direct their differentiation in 3D tissue constructs [64,69]. The main disadvantage of alginate hydrogels is the variability of their stiffness, which depends on a vast array of fabrication and formulation parameters, making it difficult to develop standardized hydrogels with reproducible mechanical and physical properties. 

### 2.3. Hyaluronic Acid

Hyaluronic acid (HA) is a linear non-sulfated glycosaminoglycan (polysaccharide) found in most tissues throughout the human body. HA is a fundamental component of the native ECM with key roles in morphogenesis, homeostasis and wound healing; it mediates matrix hydration and organization and provides both biochemical and biomechanical cues to cells. Its native role in tissues makes it an excellent bioactive material for cell culture and tissue engineering, where its biological signaling can be leveraged to direct cell behavior. For these reasons, HA is a popular material for research and has been used for several decades in mechanobiology, cell culture, tissue engineering, regenerative medicine and drug delivery.

HA presents several functional groups (carboxyl, hydroxyl and -NHCOCH_3_) in its backbone, which can be targeted to produce HA derivatives with multiple functional groups of interest [70]. The different functional modifications, in turn, offer a variety of crosslinking methods, which have been reviewed elsewhere [71]. Thiol-modified HA (HA-SH) can form a self-crosslinked hydrogel or be crosslinked via Michael-type addition with several linkers, including PEG-diacrylate (PEGDA) [72] and PEG- vinylsulfone (PG-VS) [73]. 

Similarly, methacrylated HA (MeHA) can be crosslinked via Michael-type addition with dithiothreitol (DTT) or other thiolated compounds, which allows for the tuning of mechanical properties by adjusting the proportion of DTT [74]. MeHA is also susceptible to photocrosslinking, where the final hydrogel stiffness (2–100 kPa) can be controlled by the MW, concentration and degree of methacrylation of the macromer [34]. More recently, photocrosslinking between HA-SH and MeHa via thiol-ene coupling has been explored for in situ gelation [75].

To facilitate cell adhesion, HA hydrogels are often functionalized with cell-adhesive RGD motifs, which can be readily conjugated with MeHA through Michael addition due to the presence of thiols in RGD peptides [34,74,76,77,78]. Interestingly, unmodified HA does not contain integrin binding sites, and cells do not form focal adhesions on HA substrates. However, cells can attach to and interact with HA hydrogels through a wide array of cell surface receptors and HA-binding proteins (HABPs or hyaladherins), including CD44, aggrecan and the receptor for hyaluronan mediated motility (RHAMM) [79]. However, this can result in confounding biological interactions between cells and the HA substrate.

HA hydrogels can be synthesized with a range of rigidities similar to synthetic biomaterials (1–100 kPa) either by tuning the concentration of crosslinker (e.g., MeHA with DTT [76,78]) or the concentration of macromer (MeHA photopolymerization [77]). This range of rigidities can mimic the native stiffness of different tissues and pathologies, making HA substrates suitable for mechanobiology studies. One other interesting application for HA is the development of dynamic hydrogels by combining different crosslinking methods—that is, substrates that can change their mechanical properties over time or following a user-defined trigger. This multi-step crosslinking enables spatial stiffness patterning and temporal control over the hydrogel mechanical properties [78,80]. Softening hydrogels have also been developed using hydrolysis-sensitive crosslinkers [74]. 

### 2.4. Polyethylene Glycol

Polyethylene glycol (PEG) is a synthetic polymer that has been widely used as a biomaterial in cell culture, tissue engineering and mechanobiology applications. PEG can be modified with a variety of functional groups, including carboxyl, amine, thiol and acrylate, and is available in both linear and branched formats [81]. Interestingly, PEG hydrogels are highly biocompatible, chemically inert and hydrophilic, making them resistant to protein adsorption and cell adhesion [21]. While this means that PEG hydrogels require functionalization, the low background adhesion and flexible chemistry enable control over the ligand density and presentation [81] and create interesting patterning opportunities through the use of microcontact printing [82] and other photolithography techniques [83,84]. As a result, PEG hydrogels offer a very high degree of control and flexibility compared to other synthetic biomaterials. 

Owing to the chemical versatility, PEG hydrogel networks can be assembled through a variety of crosslinking methods, including Michael-type addition [85], thiol-ene coupling [86,87] and acrylate photo-polymerization [35,88], depending on the functional groups of the modified PEG. Photopolymerization of the acrylate derivatives of PEG, such as polyethylene glycol acrylate (PEGDA) or polyethylene glycol methacrylate (PEGMA), are among the most common methods for PEG hydrogel fabrication and excel for 3D photoencapsulation. The mechanical properties of PEG hydrogels can be tuned by adjusting the crosslinking density, which can, in turn, be controlled by the type of crosslinker, the crosslinker concentration and the exposure time or dose. 

The use of photopolymerization also forms the basis for dynamic hydrogels, whose mechanical properties can be dynamically controlled through exposure to light. Both softening [89] and stiffening [90,91] hydrogels have been achieved using photodegradable or photo-crosslinked PEG, respectively, as well as switchable (reversible) hydrogels that rely on azobenzene crosslinking [92]. These dynamic hydrogels have been used in the field of mechanobiology to study mechanical memory [1].

The versatility of chemistry amenable to PEG can also be leveraged to functionalize hydrogels with a variety of motifs and proteins of interest. Cell-adhesive ligands, such as RGD or KQAGDV, and ECM-bound growth factors, such as TGF-β or EGF, can be conjugated with PEGDA through acrylate copolymerization or with PEG-VS (PEG Vinyl sulfone) via Michael-type addition. Copolymerization with diacrylate peptide derivatives enables the use of peptide-based crosslinkers that are susceptible to proteolytic degradation by MMPs and other cell-secreted enzymes [81]. 

PEG is also commonly used in combination with other biomaterials. For instance, PEG block co-polymer gels incorporating degradable polymers, such as PLA, are used in applications where biodegradability is desirable [81,93,94]. Modified PEG can also be used as a crosslinker for other biomaterials, such as HA due to its chemical flexibility and biocompatibility [72,95]. Due to the low protein adsorption and hydrophilicity, PEG has also been used as an antifouling coat or as a background material to prevent cell adhesion in micropatterning [96,97,98]. 

### 2.5. Polyacrylamide

Polyacrylamide (PAA) hydrogels are synthesized through the polymerization of acrylamide monomers in combination with a crosslinker (bisacrylamide). The stiffness of PAA hydrogels can be easily tuned over a wide range (1–1000 kPa) by adjusting the proportion of bisacrylamide (crosslinker) to acrylamide (monomer) [37]. The protocols to fabricate PAA gels of different rigidities are well established and easily accessible, which has facilitated the adoption of this biomaterial as a gold standard for mechanobiology studies. Moreover, by tuning hydrogel rigidity without affecting the hydrogel density or composition, the effect of substrate stiffness on cell behavior can be assessed independently from other chemical or physical properties. 

Similar to other synthetic hydrogels, PAA on its own does not enable cell attachment, and surface functionalization with cell adhesion ligands or proteins is required. Cell-adhesion ligands are usually covalently bound to the PAA surface using the linker sulfo-SANPAH, although other linkers have been explored, such as *N*-hydroxysuccinimide-acrylamide (NHS-AA) ester [99], hydrazine modifications [100] and 1-ethyl-3-(3-dimethylaminopropyl) carbodiimide [101]. Interestingly, this method of functionalization enables independent control of the ligand presentation and mechanical properties, and thus the effect of these two factors on cell behavior can be decoupled. A variety of ECM proteins have been used to functionalize PAA hydrogels, including collagen I, collagen IV, fibronectin and laminin [102,103,104,105]. 

PAA hydrogels of different stiffness have been instrumental in the development of mechanobiology. Stiffness-tunable PAA hydrogels have been used as 2D substrates to demonstrate the effect of stiffness on stem cell fate [106], cancer associated fibroblast activation [13], cancer cell malignancy [14] and cell migration [107,108].

### 2.6. Polydimethylsiloxane

Polydimethylsiloxane (PDMS) is another widely used synthetic polymer that produces gels with well-defined and reproducible mechanical properties. PDMS is commercially available as a two-part formulation of prepolymer + crosslinker, and the stiffness of the resulting gel can be readily tuned by adjusting the proportions of the two components, resulting in a wide range of rigidities (5 kPa to 1 MPa) [38,39,40]. In addition to the ease of tuning, PDMS is highly biocompatible, hydrophobic, and chemically inert, making it ideal as a cell culture platform. PDMS is also widely used in microfluidics and as a biomaterial in implants.

Due to its surface properties, PDMS needs to be functionalized to enable cell adhesion, although cell culture on bare PDMS has been studied in certain instances [109,110]. ECM proteins, including collagen, fibronectin and laminin, can be readily deposited on the surface of PDMS by adsorption [39,111,112,113,114]. However, this method is not stable for long term cell cultures [111]. 

Oxygen plasma treatment can be used to introduce hydroxyl groups, rendering the surface of PDMS hydrophilic and more suitable for cell culture either directly or by facilitating the deposition of ECM proteins [111], although PDMS tends to revert to its hydrophobic state. Long term protein conjugation can be achieved by functionalizing the surface of PDMS with (3-aminopropyl)triethoxysilane (APTES) after oxygen plasma treatment—a common silanization procedure to change the surface chemistry of PDMS. ECM proteins can then be deposited on the APTES functionalized PDMS (improving retention [115]) or covalently bound to the amine groups in APTES using glutaraldehyde as crosslinker [116,117].

### 2.7. Polypeptides

Polypeptide hydrogels are composed of short, well-defined oligopeptides made up of specific amino acid sequences that are designed to assemble into supramolecular structures. The short peptides self-assemble into secondary structures, such as β-sheets or α-helices, become elongated to form fibrillar structures, and when above a critical concentration these interact with each other to form a fibrous hydrogel network with highly reproducible fiber dimensions and pore size [118]. 

This design makes peptide hydrogels easy to tune and modify, as discrete changes in the amino acid sequence can yield hydrogels with a wide range of properties [41,119,120]. The control over the sequence also provides more degrees of freedom in the hydrogel design, thus, enabling the independent tuning of different parameters, including the mechanical and chemical properties, cell–matrix interactions and degradability. This flexibility in both design and tuning provides a powerful toolkit with applications beyond mechanobiology [21]. 

One other major advantage of peptide hydrogels gels is the potential for direct functionalization, that is, the incorporation of functional motifs directly in the hydrogel formulation. By relying on amino acids, peptide gels can directly integrate proteins of interest within their sequence without disrupting the self-assembly [121,122,123]. The protein of interest is, thus, synthesized or expressed along with, and directly integrated into, the hydrogel structure, eliminating the need for post-fabrication modifications and allowing for direct control over the distribution and presentation of functional motifs. Functional motifs of interests include RGD sequences and other cell-adhesive ECM proteins (e.g., fibronectin) or bioactive factors (e.g., TGF-β and BMP). The high degree of control over the structure and properties, as well as the potential for functionalization, make peptide gels very attractive for mechanobiology studies [21].

## 3. Results

### 3.1. Self-Assembling Polypeptide Matrices Remain Mechanically Stable at Acidic pH

To serve as a platform to model the tumor microenvironment, a substrate should enable independent tuning of both its stiffness and extracellular pH. One of the most widely used substrates in mechanobiology is polyacrylamide (PAA) gels [37], which became the substrate of choice in several foundational studies in the field. The stiffness of PAA hydrogels can be controlled within a large range (1–1000 kPa) by tuning the relative concentrations of acrylamide (monomer) and bisacrylamide (crosslinker), thereby, resulting in gels that can be tailored to a variety of applications. 

PeptiGels are a new family of self-assembled polypeptide hydrogels that offer tunable mechanical properties within a range similar to PAA combined with high reproducibility and biocompatibility, making them a good candidate to recapitulate the properties of the tumor microenvironment in 2D and 3D culture. They are based on β-sheet-forming peptide sequences with alternating hydrophilic and hydrophobic amino acids as originally proposed by Zhang et al. [124]. These sequences self-assemble into amphipathic β-sheets that stack in an antiparallel manner to form fibrous supramolecular structures with fiber diameters in the range of 3–5 nm [120]. 

Above a critical concentration, the fibers entangle and associate, producing a nanofibrous porous hydrogel that mimics the architecture of the native ECM. The viscoelastic mechanical properties [125] and fiber network architecture (branched vs. associated) of the hydrogel can be tuned by adjusting the amphiphilicity of the peptide, which control the interfiber interactions, without affecting the structure of individual fibers [120,126]. The system is designed to self-assemble in response to changes in ionic strength, such as the addition of cell culture media, offering ease of fabrication and 3D cell encapsulation.

**Figure 1:**
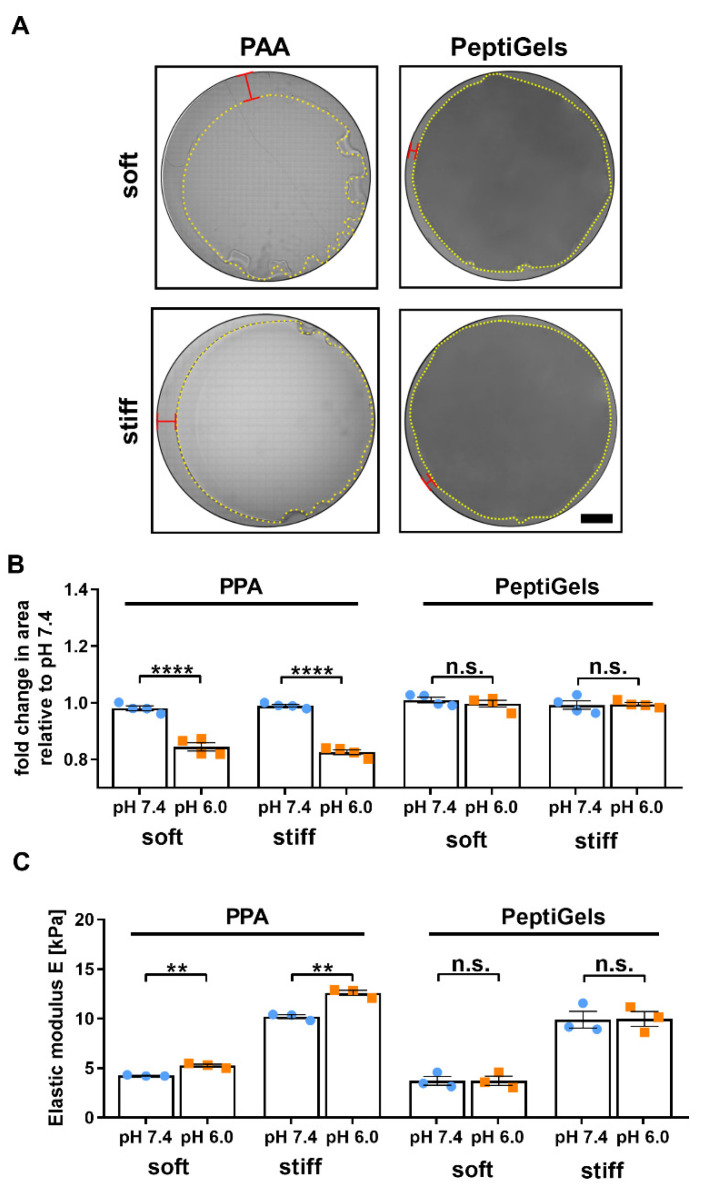
Polyacrylamide (PAA) gels (but not PeptiGels) shrank and changed mechanical properties at acidic pH. (**A**) Images of gels after adjusting the pH from 7.4 to 6.0. Yellow lines represent the perimeter of gels after shrinkage with red bracket indicating reduction in gel diameter. (**B**) Average change in gel area 24 h after placing samples in pH 7.4 or pH 6.0, normalized against pH 7.4 for each gel type. (**C**) Average elastic modulus across 0.1–10% strain calculated as E = 2 × G’ (1 + υ) where, υ = Poisson’s ratio of 0.48 for PAA and 0.5 for PeptiGels. Histogram bars represent the mean ± SEM, and dots represent average value for each experimental replicate *n* (for (**B**) *n* = 4, for (**C**) *n* = 3, three gels measured per replicate), and the scale bar is 20 mm. Markers denote a significant difference between groups labeled with brackets by *t*-test; n.s.–not significant, ** 0.001 < *p* < 0.01 and **** *p* < 0.0001.

To assess the mechanical stability of PAA gels and PeptiGels, we decided to analyze whether their mechanical properties were affected by changes in pH of the medium. We observed that both soft (4 kPa) and stiff (10 kPa) PAA gels underwent significant shrinking when subjected to pH 6.0 for 24 h, resulting in a ~20% reduction (*n* = 4) in the gel area (Figure 1A,B). Conversely, different formulations of PeptiGels (Gamma 2 and Alpha 2, corresponding to soft and stiff matrices, respectively) underwent no significant shrinking or change in surface area when subjected to pH 6.0 (*n* = 4). 

To further analyze the effect of pH on the mechanical stability of PAA gels and PeptiGels, we characterized their elastic moduli at pH 7.4 and 6.0 using oscillatory rheology (Figure 1C). Both soft and stiff PAA gels showed an increase in their Young’s Moduli at low pH, from 4.2 ± 0.1 to 5.3 ± 0.1 kPa for soft gels, and from 10.2 ± 0.2 to 12.6 ± 0.3 kPa for stiff gels, respectively (mean ± SEM, *n* = 3); a stiffening that is consistent with the shrinking previously observed. In contrast, PeptiGels showed no significant changes in mechanical properties when subjected to different pH conditions. These results indicate that PeptiGels are mechanically stable over a range of pH relevant for cancer research (pH 7.4–6.0, corresponding to physiological and tumor pH, respectively).

**Figure 2:**
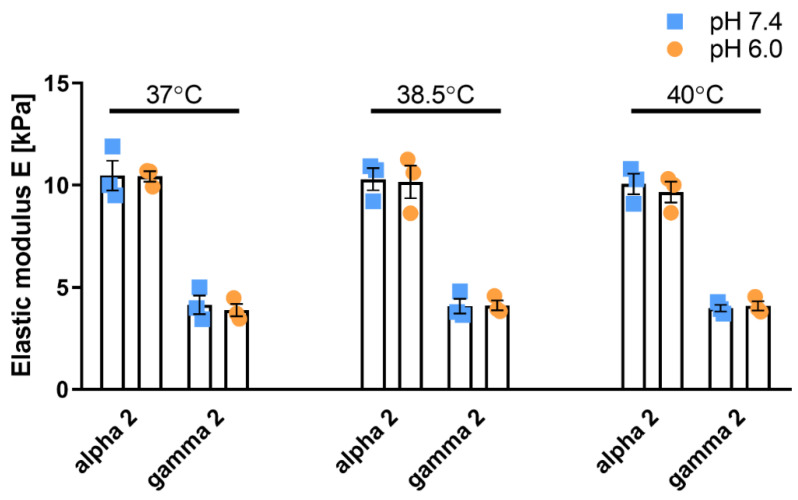
PeptiGels maintained stiffness stability in increased temperature and acidic pH. Average elastic modulus across 0.1–10% strain calculated as E = 2 × G’ (1 + υ) where, υ = Poisson’s ratio of 0.5. Histogram bars represent the mean ± SEM, and dots represent average value for each experimental replicate *n* = 3. No statistically significant difference between 37 °C, pH 7.4; 37 °C, pH 6.0; 38.5 °C, pH 7.4; 38.5 °C, pH 6.0; 40 °C, pH 7.4; and 40 °C, pH 6.0 for Alpha 2 and Gamma 2, respectively, tested with ANOVA with Dunnett’s post hoc test.

We then focused our attention on the effect of temperature on mechanical properties of the peptide gels. Tumors often present a slightly elevated temperature as a result of increased metabolism and inflammation. This increase in temperature (<41 °C) subjects cancer cells to chronic mild heat stress, which can increase the activation of heat shock proteins (chaperones) and contribute to tumorigenesis and cancer cell survival. We subjected PeptiGels to different conditions of pH (6.0 and 7.4) and temperature (37, 38.5 and 40 °C) and observed that both Gamma 2 (soft, 4 kPa) and Alpha 2 (stiff, 10 kPa) gels were stable at different pH over a temperature range between 37 °C and 40 °C with no significant changes to their mechanical properties in response to temperature or acidity (Figure 2). 

The mechanical and chemical stability of PeptiGels allow the independent tuning of the extracellular pH, temperature and substrate stiffness, thus, making them suitable for recapitulating the hallmark characteristics of the tumor microenvironment. For this reason, we decided to use these matrices as a cell culture platform to study the combined effect of pH, temperature and stiffness on cancer cell proliferation, apoptosis and signaling.

### 3.2. Extracellular pH and Matrix Stiffness Regulate Cancer Cell Proliferation

**Figure 3:**
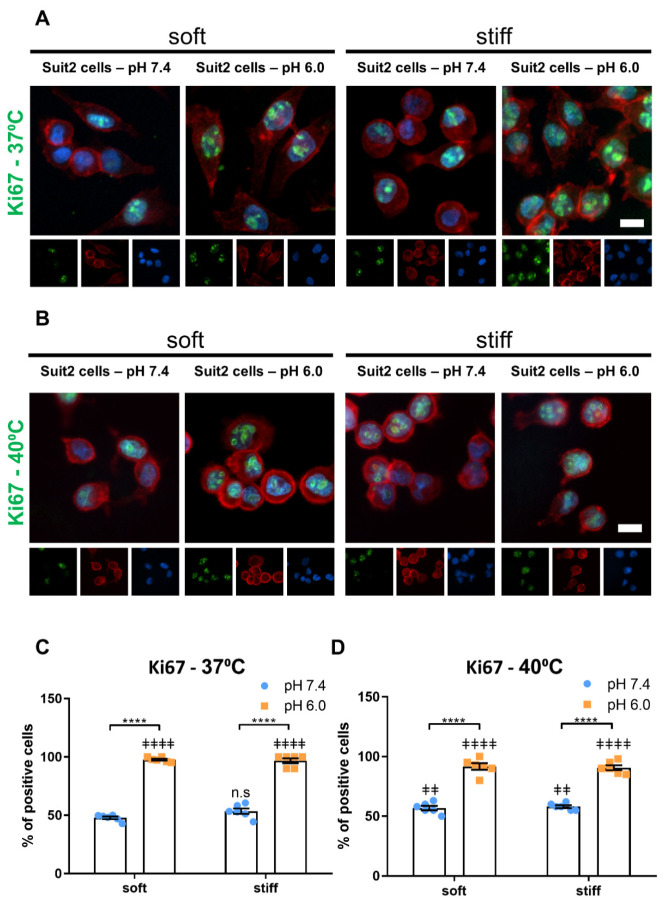
Low, tumor-mimicking pH increased the proliferation of Suit-2 007 cells cultured on soft and stiff self-assembling peptide hydrogels. (**A**,**B**) Widefield, epifluorescent images of Ki67 as a marker of proliferation for cells cultured on soft (Gamma 2) and stiff (Alpha 2) Manchester BIOGEL PeptiGels in physiologically healthy (7.4) and tumor-mimicking (6.0) pH at 37 °C (**A**) and 40 °C (**B**). Ki67 (green), actin (red) and nucleus (blue). Scale bar represents 50 µm. (**C**,**D**) Percentage of Ki67 positive nuclei presented in (**A**,**B**), respectively. Histogram bars represent the mean ± SEM, and dots represent the average percentage of Ki67 positive cells per individual sample, across three experimental replicates. Three experimental replicates. * markers denote a significant difference between bracket-marked groups by *t*-test, **** *p* < 0.0001; ‡ markers denote a significant difference from soft pH 7.4 condition by ANOVA with Dunnett’s post hoc test; n.s.–not significant, ‡‡ 0.001 < *p* < 0.01 and ‡‡‡‡ *p* < 0.0001.

The development of cell culture platforms that can recapitulate the properties of the tumor microenvironment (TME) is key to improving the current in vitro models of cancer. Two widespread characteristics of the TME are a low pH, arising from a reliance on glycolytic metabolism, and high matrix stiffness, resulting from the remodeling of the tumor stroma by cancer associated fibroblasts [11,127,128]. In order to understand how these microenvironmental factors direct cancer cell behavior, we cultured Suit2-007 cells, a pancreatic ductal adenocarcinoma (PDAC) cell line, on PeptiGel matrices of different rigidity (soft–4 kPa or stiff–10 kPa) and under different conditions of pH, representing physiological pH (7.4) and tumor acidosis (6.0). Then, to monitor the effect of these two microenvironmental factors, we analyzed the expression of Ki67, a common proliferation marker and a prognosis marker for cancer (Figure 3A,C) [129,130,131,132].

In soft (Gamma 2) PeptiGels, we observed an increase in the percentage of Suit2-007 cells expressing Ki67 (Figure 2), from 48 ± 1% at pH 7.4 (mean ± SEM, *n* = 6) to 98 ± 1% at pH 6.0 (mean ± SEM, *n* = 6). A similar trend was observed in stiff (Alpha 2) PeptiGels, increasing from 53 ± 2% at pH 7.4 to 97 ± 2% at pH 6.0 (mean ± SEM, *n* = 6). On the other hand, the substrate stiffness was found to have no significant effect on the expression of Ki67 in Suit2 cells at either of the two pH conditions. These results indicate that low extracellular pH, such as is found in tumors, promotes cancer cell proliferation. 

We then conducted the same experiment at 40 °C to assess the impact of temperature on the regulation of cell proliferation by pH and temperature (Figure 3B,D). We observed a similar trend with an increase in Ki67 expression between pH 7.4 and pH 6.0 in both soft and stiff PeptiGels. In soft (Gamma 2) gels, we observed an increase in proliferation at 40 °C with respect to 37 °C at pH 7.4 (from 47.8 ± 1.1% to 56.8 ± 1.9%, mean ± SEM, *n* = 6, *p* < 0.01, Mann–Whitney test), whereas temperature was found to have no significant effect on the stiff gels at either pH condition. Interestingly, we found that, on soft gels at pH 6.0, the Ki67 expression decreased at 40 °C with respect to 37 °C (*n* = 6, *p* < 0.05, Mann–Whitney test), thus, suggesting that a combination of acidic pH and high temperature might have deleterious effects on cancer cells. 

### 3.3. Extracellular pH and Matrix Stiffness Regulate Cancer Cell Apoptosis

**Figure 4:**
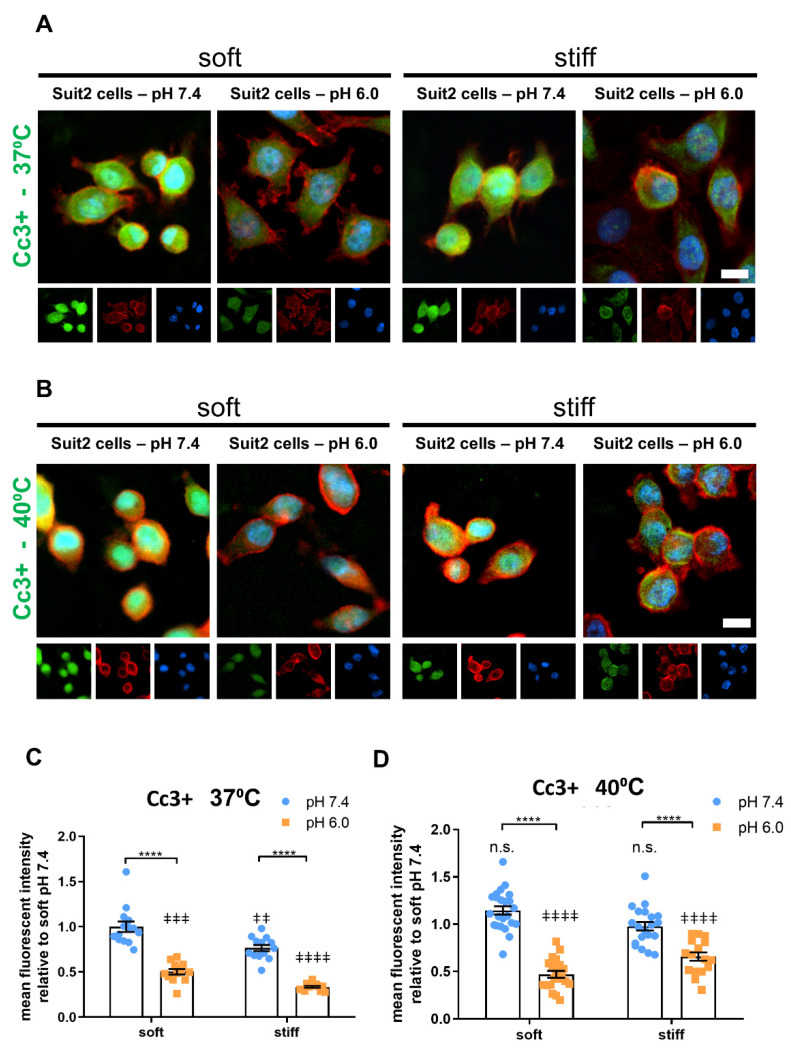
Apoptosis of Suit-2 007 cells is decreased as a result of tumor pH (6.0) and higher stiffness. (**A**,**B**) Widefield, epifluorescent images of Cc3+ (Cleaved caspase 3) as a marker of apoptosis, for cells cultured on soft (Gamma 2) and stiff (Alpha 2) Manchester BIOGEL PeptiGels in physiologically healthy (7.4) and tumor-mimicking (6.0) pH at 37 °C (**A**) and 40 °C (**B**). Cc3+ (green), actin (red) and nucleus (blue). Scale bar represents 50 µm. (**C**,**D**) MFI-mean fluorescence intensity (expressed in arbitrary units) of Cc3+ stained cells presented in (**A**,**B**), respectively. Histogram bars represent the mean ± SEM, and dots represent the average Cc3+ intensity within a sample (20 cells measured per sample) across three experimental replicates. * markers denote a significant difference between bracket-marked groups by *t*-test, **** *p* < 0.0001. ‡ markers denote a significant difference from soft pH 7.4 condition by ANOVA with Dunnett’s post hoc test; ‡‡ 0.001 < *p* < 0.01, ‡‡‡ 0.0001 < *p* < 0.001 and ‡‡‡‡ *p* < 0.0001.

We then set to investigate the effect of extracellular pH and substrate stiffness on cancer cell apoptosis. To this end, we characterized the expression of cleaved caspase 3 (Cc3+) on Suit2 cells via immunofluorescence. In both soft (Gamma 2) and stiff (Alpha 2) PeptiGels, we observed a decrease in the relative mean fluorescence intensity of Cc3+ between pH 7.4 (1.00 ± 0.06 for soft gels, 0.77 ± 0.03 for stiff gels; mean ± SEM, *n* = 14 and 13 for soft and stiff, respectively) and pH 6.0 (0.50 ± 0.03 for soft gels and 0.33 ± 0.01 for stiff gels; mean ± SEM, *n* = 13), indicating a decrease in the apoptosis rate with acidic pH (Figure 4A,C). 

Interestingly, at both pH 7.4 and 6.0, we observed that cells cultured on stiff gels displayed a lower Cc3+ expression (i.e., a lower apoptosis rate) compared with the cells cultured on soft gels at the same pH (*p* < 0.001, Mann–Whitney test), suggesting that mechanical stimuli from the stiff substrate promoted cancer cell survival and that both extracellular pH and substrate rigidity contributed to the regulation of cancer cell apoptosis. 

We then conducted the same experiments at 40 °C to analyze the effect of mild heat stress on cancer cell apoptosis as well as its interaction with pH and stiffness (Figure 4B,D). In all the conditions analyzed here, we observed an increase in Cc3+ expression at 40 °C relative to the same conditions of pH and stiffness at 37 °C, except for soft (Gamma 2) gels at pH 6.0. This effect was more significant on stiff PeptiGels at pH 6.0 (the conditions that more closely resemble the tumor microenvironment), where we observed a two-fold increase in apoptosis between 37 and 40 °C (Mann–Whitney test, *p* < 0.0001, *n* = 13 and 17 for 37 and 40 °C, respectively). 

These results are consistent with an increase in apoptosis caused by heat stress. Taken together with the upregulation of proliferation by pH and temperature, these results suggest that microenvironmental factors play an important role in regulating cancer cell proliferation and survival and highlight the importance of recapitulating the wider extracellular context of the tumor microenvironment.

### 3.4. Extracellular pH, Temperature and Matrix Stiffness Regulate YAP-1 and HIF-1A Signaling

The Yes-associated protein 1 (YAP-1) is a transcription factor that plays a fundamental role in cellular mechanotransduction. YAP-1 is activated downstream of a variety of mechanical stimuli, most notably high substrate stiffness, and coordinates the cell’s mechanical activity. While inactive, YAP-1 remains cytoplasmic; however, it translocates to the nucleus when activated where it carries out its function as a transcription factor to regulate the expression of a variety of genes, including connective tissue growth factor (CTGF) and Ankyrin-1 (ANKRD1). 

**Figure 5:**
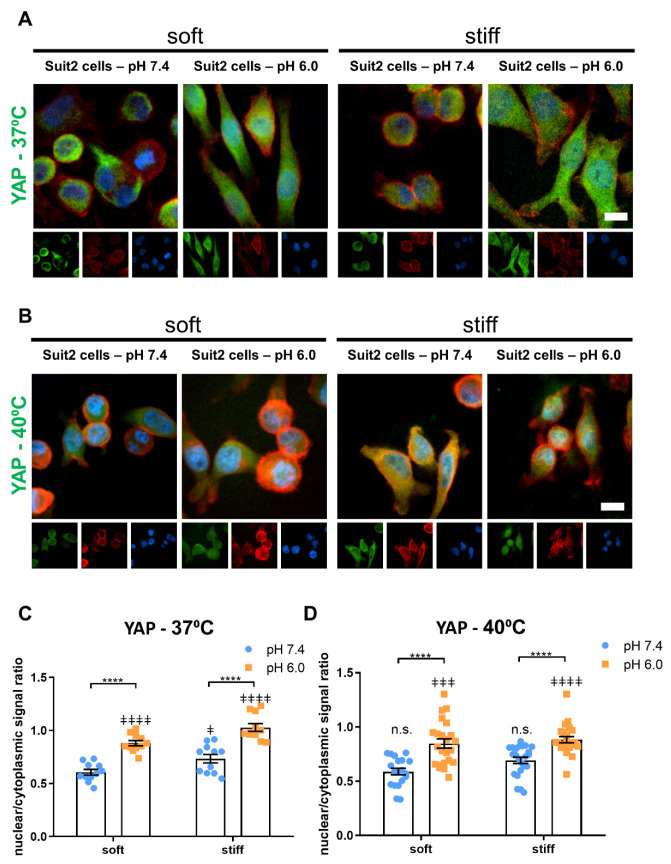
Acidic pH (6.0) of culture media and tumor-mimicking stiffness of the substrate increase nuclear translocation of YAP in Suit-2 007 cells. (A-B) Widefield, epifluorescent images of YAP (Yes-associated protein) for cells cultured on soft (Gamma 2) and stiff (Alpha 2) Manchester BIOGEL PeptiGels in physiologically healthy (7.4) and tumor-mimicking (6.0) pH at 37 °C (**A**) and 40°C (**B**). Protein of interest (green), actin (red) and nucleus (blue). Scale bar represents 50 µm. (**C**,**D**) Nuclear/cytoplasmic signal ratio assessed by measuring the MFI-mean fluorescence intensity (expressed in arbitrary units) of YAP-stained cells presented in (**A**,**B**), respectively. Histogram bars represent the mean ± SEM, and dots represent the average yap nuclear/cytoplasm ratio within a sample (20 cells measured per sample) across three experimental replicates. * markers denote a significant difference between bracket-marked groups by *t*-test, **** *p* < 0.0001. ‡ markers denote a significant difference from soft pH 7.4 condition at 37 °C by ANOVA with Dunnett’s post hoc test; ‡ 0.01 < *p* < 0.05, ‡‡‡ 0.0001 < *p* < 0.001 and ‡‡‡‡ *p* < 0.0001.

Using immunofluorescence, we characterized the nuclear to cytoplasmic ratio of YAP in Suit2 cells cultured on different conditions of substrate rigidity and pH (Figure 5A,C). The nuclear to cytoplasmic ratio increased from 0.60 ± 0.03 at pH 7.4 to 0.88 ± 0.03 at pH 6.0 in soft substrates and from 0.73 ± 0.04 at pH 7.4 to 1.03 ± 0.04 at pH 6.0 in stiff substrates (mean ± SEM, *n* = 11). Moreover, cells cultured on stiff gels displayed a higher nuclear to cytoplasmic YAP ratio compared with those cultured on soft gels at a given pH (*p* < 0.05 at pH 7.4 and *p* < 0.01 at pH 6.0, Mann–Whitney Test, *n* = 11), consistent with the activation of YAP via mechanotransduction that has been previously observed.

The upregulation of YAP-1 downstream genes CTGF and ANKRD1 by substrate stiffness has been reported by our group and others. Here, we analyzed the effect of pH on the expression of CTGF and ANKRD1 at the mRNA via RT-qPCR and observed a similar upregulation at acidic pH compared to pH 7.4 (Figure S1). These results indicate that low pH can activate YAP independently from the substrate stiffness and suggest that both extracellular pH and stiffness contribute to the activation of YAP and its downstream genes in cancer. 

At 40 °C, we observed a similar trend for the nuclear/cytoplasmic YAP ratio as a function of substrate stiffness and pH (Figure 5B,D), with no significant differences between each condition at 40 °C and the corresponding condition at 37 °C, except for Suit2 cells cultured on stiff (Alpha 2) gels at pH 6.0, where we found a slight decrease in the nuclear/cytoplasmic YAP ratio, from 1.03 ± 0.04 at 37 °C to 0.88 ± 0.03 at 40 °C (mean ± SEM, *n* = 11 and 23 samples for 37 and 40 °C, respectively). 

HIF-1A is another master regulatory switch that plays a central role in cancer progression by controlling broad signaling pathways involved in metabolism, survival and angiogenesis. Its expression correlates with increased malignancy and poor prognosis, and it represents an important therapeutic target. While the mechanisms of HIF-1A regulation by hypoxia in cancer are well understood, the role of other microenvironmental factors in modulating HIF-1A has not been characterized. Here, we analyzed the expression of HIF-1A by suit2 cells cultured in PeptiGels of different rigidity (soft and stiff) and pH (7.4 and 6.0) using immunofluorescence. 

**Figure 6:**
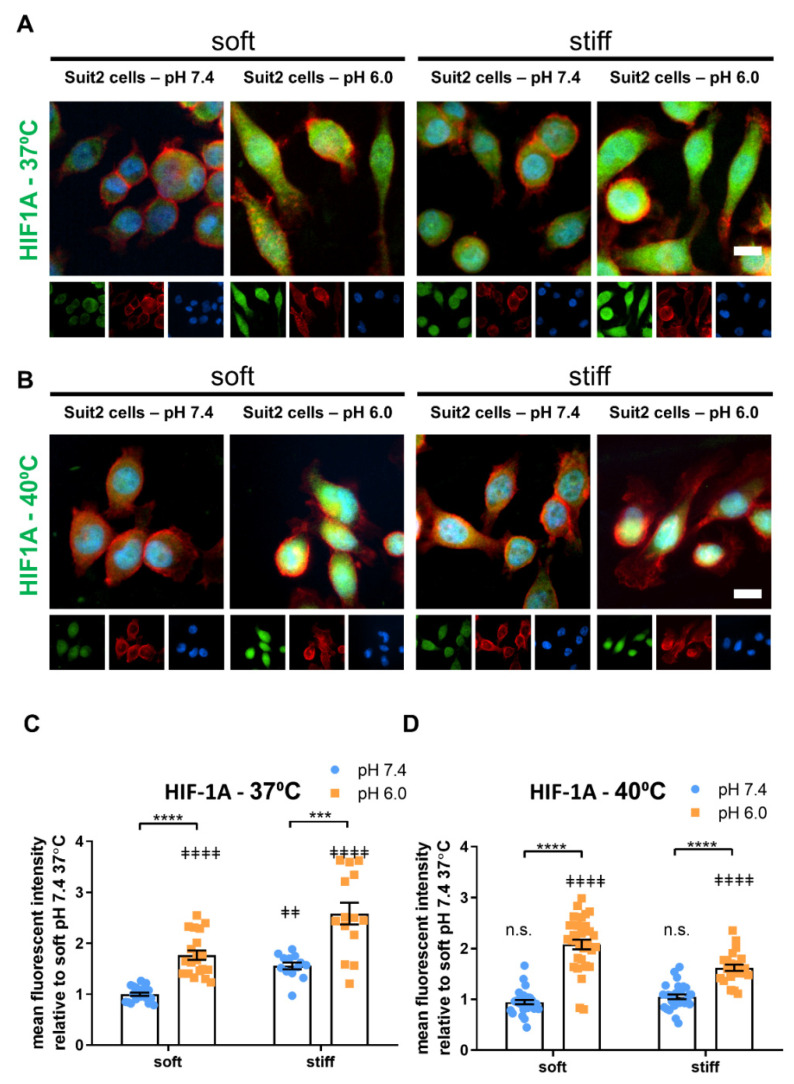
Acidic pH (6.0) of culture media and tumor-mimicking stiffness of the substrate increase the expression of Hypoxia Inducible Factor 1 Alpha in Suit-2 007 cells. (**A**,**B**) Widefield, epifluorescent images of HIF1A for cells cultured on soft (Gamma 2) and stiff (Alpha 2) Manchester BIOGEL PeptiGels in physiologically healthy (7.4) and tumor-mimicking (6.0) pH at 37 °C (**A**) and 40 °C (**B**). Protein of interest (green), actin (red) and nucleus (blue). Scale bar represents 20 µm. (**C**,**D**) Protein expression measured as the MFI-mean fluorescence intensity (expressed in arbitrary units) of HIF1A-stained cells presented in (**A**,**B**), respectively. Histogram bars represent the mean ± SEM, and dots represent the average yap nuclear/cytoplasm ratio within a sample (20 cells measured per sample) across three experimental replicates. * Markers denote a significant difference between bracket-marked groups by *t*-test, *** 0.0001 < *p*< 0.001 and **** *p* < 0.0001. ‡ markers denote a significant difference from soft pH 7.4 condition at 37 °C by ANOVA with Dunnett’s post hoc test; n.s not significant, ‡‡ 0.001 < *p* < 0.01, and ‡‡‡‡ *p* < 0.0001.

We observed that, in both soft and stiff gels, the HIF-1A expression increased at low pH (Figure 6A,C), from 1.00 ± 0.03 to 1.77 ± 0.09 in soft gels and from 1.56 ± 0.07 to 2.59 ± 0.20 in stiff gels (mean ± SEM, *n* = 20, 20, 13 and 14 in soft 7.4, soft 6.0, stiff 7.4 and stiff 6.0, respectively). Conversely, when we studied the expression of HIF-1A using qPCR, we found no significant difference at the mRNA level between pH 7.4 and pH 6.0 (Figure S2), suggesting that the regulation of HIF-1A by pH occurs at the protein level.

We then studied the effect of temperature (40 °C) on the expression of HIF-1A on Suit2 cells subjected to the same conditions of pH and substrate stiffness (Figure 6B,D) and observed a similar trend to those cultured at 37 °C. In both soft and stiff PeptiGels at 40 °C, the cells showed a higher expression of HIF-1A at pH 6.0 (2.08 ± 0.09 and 1.62 ± 0.06 for soft and stiff gels, respectively, mean ± SEM, *n* = 31 and 23 for soft and stiff respectively) compared to pH 7.4. (0.95 ± 0.04 and 1.05 ± 0.05 for soft and stiff gels, respectively, mean ± SEM, *n* = 28 for both groups). However, we found that, in stiff gels (at both pH 7.4 and pH 6.0), the expression of HIF-1A decreased with the increase in temperature (40 °C) with respect to the same conditions of pH and stiffness at 37 °C (for pH 7.4, *p* < 0.0001, *n* = 13 and 28 for 37 and 40 °C, respectively; and for pH 6.0, *p* < 0.001, *n* = 14 and 23 for 37 and 40 °C, respectively. Mann–Whitney test). These results indicate that mild heat stress antagonized the upregulation of HIF-1A by acidic pH and stiffness.

## 4. Discussion

Tunable cell culture platforms are a fundamental research tool in fields as diverse as cell and developmental biology, mechanobiology, tissue engineering and cancer research. The selection of the substrate biomaterial often results in a tradeoff between flexibility, ease of tuneability and 3D scalability. Here, we assessed the suitability of a self-assembling peptide hydrogel as a cell culture platform to recapitulate the conditions of the tumor microenvironment: low extracellular pH, high substrate stiffness and elevated temperature. Compared to PAA, we found that the mechanical properties of this gel were not affected by the pH or temperature of the medium, allowing us to tune the extracellular pH, stiffness and temperature independently while maintaining the other factors as constant. 

By tuning the different culture parameters, it is possible to analyze both the independent contribution of different factors as well as their interactions in order to develop a broader picture of the role of the tumor microenvironment on cancer cell behavior. Self-assembling peptide hydrogels are amenable to 3D scalability, contrary to synthetic substrates, like PAA and PDMS, thus, facilitating translation between 2D and 3D models. However, more work is required to understand the interaction between cells and fibrous peptide networks. 

Here, we leveraged the stability and tunability of a peptide hydrogel to explore the response of pancreatic cancer cells to different combinations of pH, temperature and substrate stiffness. We found that a low pH increased proliferation and reduced apoptosis, in line with previous studies that analyzed the effect of extracellular pH on other cancer cell lines [133,134,135]. Consistent with previous reports, we found that mild heat stress (40 °C) increased cell apoptosis. 

However, while we did not find the substrate stiffness or pH to have a protective effect on heat stress, cells subjected to the conditions that more closely resembled the tumor microenvironment (stiff substrate, pH 6.0, 40 °C) still displayed lower levels of Cc3+ (apoptosis) compared with those under physiological conditions (soft substrate, pH 7.4, 37 °C), indicating that the tumor microenvironment had a net positive effect on cancer cell survival. These results illustrate the importance of considering multiple microenvironmental factors when designing cancer models, as different culture parameters can have synergistic or antagonistic effects on different aspects of cell behavior. 

We found that the substrate stiffness, temperature and pH all positively regulated HIF-1A at the protein level. We previously reported that high substrate stiffness upregulated HIF-1A at the mRNA level [136]. Here, we found that extracellular pH had no significant effect on HIF-1A mRNA. Under physiological conditions, HIF-1A is regulated through its canonical oxygen-dependent pathway. HIF-1A is continuously produced; however, in normoxia, the proteins prolyl hydroxylase (PHD) and von Hippel–Lindau (VHL) act in tandem to ubiquitinate HIF-1A, tagging it for proteasomal degradation. In the absence of oxygen (hypoxia), this mechanism of degradation is inhibited, resulting in rapidly increasing HIF-1A levels. It is possible that low pH (6.0) similarly impairs the mechanism of HIF-1A degradation by PHD and VHL, thereby, increasing its protein levels without affecting mRNA expression. 

While HIF-1A expression in cancer has been studied in the context of its metabolism and hypoxia, here, we found that matrix stiffness could also modulate its expression independently from the oxygen availability. The regulation of HIF-1A by mechanical stimuli has been previously reported in the vasculature, where HIF-1A expression was induced in endothelial cells by low wall shear stress [137] and in cardiomyocytes by stretching [138]. Our group also reported that HIF-1A expression was reduced by tamoxifen, a drug known to inhibit cell contractility and mechanosensing [136]. 

This mechanism of mechano-regulation of HIF-1A by matrix stiffness represents an unexplored angle to inhibit HIF-1A expression and could provide novel therapeutic targets. By regulating HIF-1A, this mechanism could provide a link between cancer mechanotransduction and metabolism as well as a potential pathway for mechanical cues from the pre-malignant ECM to drive metabolic changes in epithelial cells during the early stages of cancer evolution. However, more work will be required to identify the mechanisms of regulation and elucidate the signaling pathway. 

## 5. Materials and Methods 

### 5.1. Cell, Reagents, and Antibodies

Suit2-007 cells were kindly donated by Prof. Malte Buchholz from Philipps-Universität Marburg. Cells were maintained in Dulbecco’s Modified Eagle’s Medium-low glucose (Cat. No. D5546, Sigma Aldrich, Dorset, UK) supplemented with 10% *v*/*v* FBS (Cat No. F7524, Sigma Aldrich, Dorset, UK), l-glutamine (Cat No. G7513, Sigma Aldrich, Dorset, UK), 1% *v*/*v* penicillin/streptomycin (Cat. No. P4333 Sigma Aldrich, Dorset, UK) and 1% *v*/*v* Fungizone/amphotericin B (Cat. No. 15290-026 Gibco, Carlsbad, CA, USA). Coverslips (Cat No. 631-0149P, cover glasses, 13 mm diameter, thickness No.1, VWR, Radnor, PA, USA) were coated with Manchester BIOGEL.

Peptide gels (PeptiGel Gamma 2 and PeptiGel Alpha 2, Manchester BIOGEL, Alderley Park, Cheshire, UK) were incubated in media for 1 h and coated with 10 µg/mL of Fibronectin (Cat No. PHE0023, Gibco, Carlsbad, CA, USA) in PBS (Cat. No. D8537, Sigma Aldrich, Dorset, UK) for 45 min at 37 °C. The cells were collected and counted using a hemocytometer and seeded on the peptide gels (10,000 cells per gel). The cells were cultured for 24 h in medium with the pH adjusted to 7.4 following a 24-h incubation with pH 7.4 or 6.0 in 37 or 40 °C incubation for the final 2 h.

The primary antibodies used in the experiments were YAP (SanCat. No. sc101199, Santa Cruz Biotechnology, Dallas, TX, USA, 1/200), Caspase-3 (Cat. No. ab13847, Abcam, Cambridge, UK, 1/100), Ki67 (Cat. No. 14-5698-82, ThermoFisher, Carlsbad, CA, USA, 1/100) and HIF1A (Cat. No. ab2185, Abcam, Cambridge, UK, 1/100). The secondary antibodies and dyes used in the experiments were anti-mouse IgG (H + L) Alexa-488 (Cat. No. A11029, Invitrogen, Carlsbad, CA, USA, 1/400), anti-rabbit IgG (H + L) Alexa-488 (Cat. No. A11034, Invitrogen, Carlsbad, CA, USA, 1/400), anti-rat IgG (H + L) Alexa-488 (Cat. No. A11006, Invitrogen, Carlsbad, CA, USA, 1/400) and Alexa Fluor™ 546 Phalloidin (Cat. No. A22283, Invitrogen, Carlsbad, CA, USA, 1/400).

### 5.2. Gel Preparation and Gel Contraction Assay

PeptiGels (Manchester BIOGEL, Alderley Park, Cheshire, UK) were used as supplied, and layers were prepared as described above. For the polyacrylamide gels, fabrication coverslips were covered with 3-(trimethoxysilyl) propyl methacrylate (Cat No. 440159, Sigma, Dorset, UK), incubated at room temperature for 5 min, washed in dH_2_O and left to dry at room temperature. Polyacrylamide gels of 4 and 10 kPa were prepared according to the protocol adapted from [139]. A working solution of PBS, acrylamide/bis-acrylamide (29:1) 40% vol (Cat. No. A7802, Sigma, Dorset, UK), TEMED (Cat. No. T9281, Sigma) and 10% ammonium persulfate were mixed at concentrations to achieve varying gel stiffness. 

A small drop of this working solution was applied to activated coverslips, which were placed face down on hydrophobic, dichlorodimethylsilane (Cat. No. 440272, Sigma, Dorset, UK) treated glass microscope slides and left to polymerize at room temperature for 45 min. Both gels were prepared to reach the coverslip edges, then coverslips were placed in a 24-well plate. After, the fabrication gels were incubated in the pH7.4 cell culture media for 24 h, followed by 24 h incubation in pH 6.0 or pH 7.4 cell culture media. Subsequently, the samples were imaged using brightfield with DIC. The change in area was calculated relative to the initial area of the respective coverslips.

### 5.3. Rheometry

The PeptiGel samples for stiffness measurements were prepared as described above. Polyacrylamide gels were prepared on dichlorodimethylsilane treated coverslips to improve the gel detachment. The stiffness at varying temperatures and pH values was assayed using an ar2000ex rheometer (TA Instruments, New Castle, DE, USA). Gels were loaded onto the device and measured under a strain sweep of 0.1–10%. The elastic modulus was calculated as E = 2 × G’ (1 + υ) where υ = Poisson’s ratio of 0.48 for PAA and 0.5 for PeptiGels.

### 5.4. Immunofluorescence Staining

Cell immunofluorescence staining was done on coverslips with PeptiGels coated with 10 μg/mL fibronectin in PBS (Cat. No. PHE0023, Gibco, Carlsbad, CA, USA). Following pertinent treatment, the cells were fixed with 4% *w*/*v* paraformaldehyde (Cat. No. P6148, Sigma, Dorset, UK) in D-PBS (Cat. No. D8537 Sigma, Dorset, UK) for 10 min, permeabilized with 0.1% *w*/*v* saponin (×100-100ML, Sigma, Dorset, UK) and then blocked with 1% *w*/*v* BSA (Cat. No. A8022 Sigma, Dorset, UK) and 22.52 mg/mL glycine (Cat. No. G8898, Sigma, Dorset, UK) in PBST for 30 min. After blocking, the cells were incubated with primary antibodies prepared in blocking solution overnight at 4 °C in a humidified chamber. Then, the cells were washed in D-PBS and incubated with Alexa Fluor 488-conjugated secondary antibodies and phalloidin prepared in PBS for 1 h at room temperature. Finally, the coverslips were washed in PBS and mounted in mounting reagent with 4,6-diamidino-2-phenylindole (Cat. No. P36931, Invitrogen, Carlsbad, CA, USA). 

### 5.5. Immunofluorescence Imaging Analysis

Widefield fluorescent images were taken with a Nikon Ti-e Inverted Microscope (Ti Eclipse, C-LHGFI HG Lamp, CFI Plan Fluor 40 × NA 0.6 air objective; Nikon Europe, Amsterdam, Netherlands; Neo sCMOS camera; Andor, Belfast, UK) with NIS elements AR software. The staining intensity was measured in Fiji [140] using the “mean gray value” parameter applied to a region of interest (ROI) created for manually segmented cells based on DIC images. Mean gray values for each image’s background were subtracted for each measured staining intensity. Images for DAPI were obtained in order to visualize the nucleus for the quantification of YAP staining regions. 

Nuclear ROIs were defined through automated thresholding of the DAPI channel in ImageJ. Measurements of the YAP fluorescence intensity in the nucleus were obtained in ImageJ (measured mean grey value) using the nuclear ROI (colocalization with DAPI) and compared against the cytoplasmic YAP staining intensity (measured mean grey value) for the whole cell ROI with subtracted nuclear ROI. Ratios of the nuclear to cytoplasm fluorescence intensities were calculated in order to analyze the localization of YAP in the different cells.

### 5.6. qPCR

The total RNA was extracted using the RNeasy Mini kit (Cat. No. 74104, Qiagen, Hilden, Germany), and 1 μg of total RNA was reverse-transcribed using the High-Capacity RNA-to-cDNA kit (Cat. No. 4387406Applied Biosystems, Carlsbad, CA, USA) according to the manufacturer’s instructions. qPCR was performed using the SYBR Green PCR Master Mix (Cat. No. 4309155, Applied Biosystems, Carlsbad, CA, USA) with 100 ng cDNA input in 20 μL of reaction volume. The RPL0 (60S acidic ribosomal protein) expression level was used for normalization as a housekeeping gene. 

The primer sequences were as follows: RPLP0: forward, 5′-CGGTTTCTGATTGGCTAC-3′, RPLP0: reverse, 5′-ACGATGTCACTTCCACG-3′; CTGF: forward, 5′-TTAAGAAGGGCAAAAAGTGC-3′ and reverse, 5′-CATACTCCACAGAATTTAGCTC-3′; ANKDR1: forward, 5′-TGAGTATAAACGGACAGCTC-3′ and reverse, 5′-TATCACGGAATTCGATCTGG-3′; and HIF1A: forward, 5′-AAAATCTCATCCAAGAAGCC-3′ and reverse: 5′-AATGTTCCAATTCCTACTGC-3′; All primers were used at a 300 nM final concentration. The relative gene expression was analyzed by the comparative 2–ΔΔCt method.

### 5.7. Statistical Analysis

All statistical analyses were conducted with the Prism software (version 8, GraphPad). Data were generated from multiple repeats of different biological experiments to obtain the mean values and SEM displayed throughout. *p* values were obtained through the Mann–Whitney test on unpaired samples with parametric tests used for data with a normal distribution. ANOVA and the post hoc Dunnett’s test were used to perform a multiple comparison test on normally distributed data, and the Kruskal–Wallis test was used for the multiple comparison of non-normally distributed data. Significance was set at *p* < 0.05 where graphs show significance through symbols (*/‡ 0.01 < *p* < 0.05; **/‡‡ 0.001 < *p* < 0.01; ***/‡‡‡ 0.0001 < *p* < 0.001; and ****/‡‡‡‡ *p* < 0.0001).

## 6. Conclusions

The development of novel in vitro models of cancer requires biomaterial substrates that can recapitulate the properties of the tumor microenvironment, including the substrate stiffness, acidic pH, and elevated temperature. Polyacrylamide gels, the standard choice for mechanobiology studies, do not allow for independent tuning of these culture parameters. Here, we demonstrate that self-assembling polypeptide (PeptiGel) hydrogels are a suitable platform to culture cancer cells under different conditions of pH, stiffness and temperature and to analyze the effect of these microenvironmental factors on the proliferation, apoptosis and signaling of Suit2 cells, a pancreatic cancer cell line.

## Figures and Tables

**Table 1:** Summary of biomaterials for mechanobiology substrates.

Material	Stiffness Range	Advantages	Disadvantages	References
Collagen	10–100 Pa ~1 kPa *	Mimics the physiological ECM Suitable for 3D fibrous matrices	Low stiffness range Stiffness and ligands cannot be controlled independently	[26,27,28,29,30]
Alginate	0.2–550 kPa	Excellent bioprinting properties Ionic and/or covalent crosslinking	Stiffness difficult to tune independently	[31,32,33]
Hyaluronic Acid	2–100 kPa	Multiple functional groups and crosslinking methods Dynamic hydrogels	Confounding biological signaling Requires chemical expertise	[34]
PEG	2–1000 kPa ** 10–400 kPa	High degree of design flexibility	Requires expertise to synthesize and functionalize	[35,36]
PAA	1–1000 kPa	Easy to tune over a large range of rigidity. Independent control of stiffness and ligand presentation	Not suitable for 3D culture	[37]
PDMS	5–2000 kPa	Easy to tune over a large range of rigidity. Chemically inert	Difficult to functionalize for long term culture Not suitable for 3D culture	[38,39,40]
Peptides	0.1–120 kPa **	High design flexibility Direct functionalization Highly reproducible	Early development	[41,42,43]

* Rigidities in the range of 1 kPa and higher can be obtained through additional chemical crosslinking. High stiffness sponges (100 kPa) can be achieved by freeze drying [44]. ** Storage Modulus [36,41,42,43].

## Data Availability

All data included in this manuscript will be available from authors.

## Supplementary Materials

The following are available online at https://www.mdpi.com/article/10.3390/cancers13133286/s1, Figure S1. Expression (RT qPCR) of YAP downstream genes (ANKRD1 and CTGF) at pH 7.4 and 6.0. Figure S2. Expression of HIF-1A mRNA (RT qPCR) at pH 7.4 and 6.0.
