# Supplementary material for: Self-Assembling Polypeptide Hydrogels as a Platform to Recapitulate the Tumor Microenvironment
Source: Cancers (Basel). 2021 Jun 30;13(13):3286. doi: 10.3390/cancers13133286 (PMC8267709; doi:10.3390/cancers13133286)
Supplement: Supplementary file 1 [file cancers-13-03286-s001.zip › cancers-1269814-supplementary.pdf]

cancers

MDPI

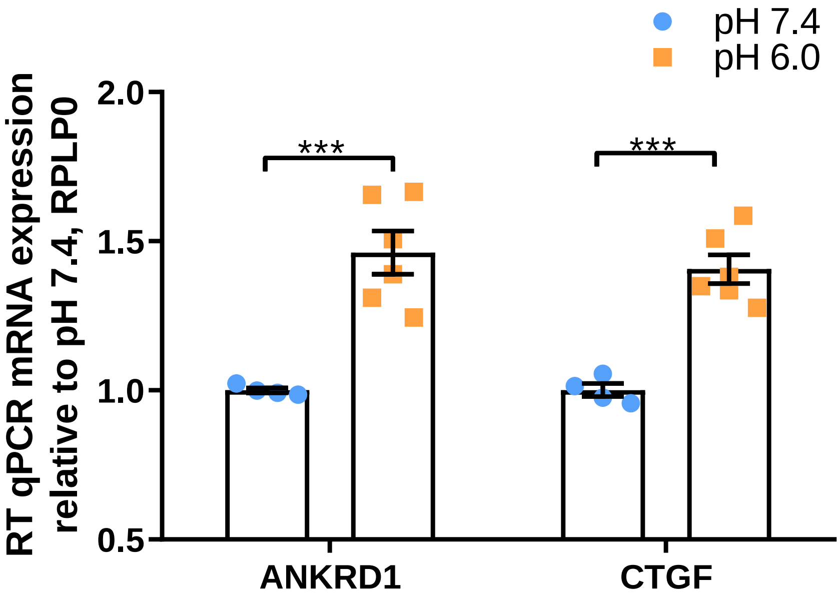

Figure S1. Expression (RT qPCR) of YAP downstream genes (ANKRD1 and CTGF) at pH 7.4 and 6.0. Acidic pH (6.0) of culture media increases the mRNA expression of YAP downstream genes in Suit-2 cells. Real time quantitative PCR ANKRD1 and CTGF mRNA expression across 4 (pH 7.4) and 6 (pH 6.0) experimental replicates. * markers denote significant difference between bracket-marked groups by t-test, *** 0.0001< p <0.001.

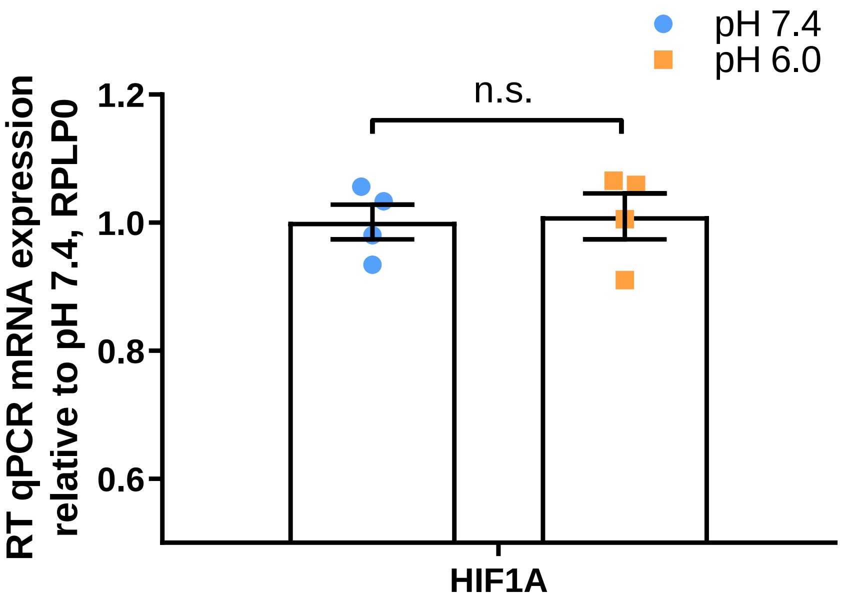

Figure S2. Expression of HIF-1A mRNA (RT qPCR) at pH 7.4 and pH 6.0. Acidic pH (6.0) of culture media does not affect the mRNA expression of HIF1A in Suit-2 cells. Real time quantitative PCR HIF1A mRNA expression across 4 (pH 7.4) and 4 (pH 6.0) experimental replicates. Markers denote significant difference between pH 7.4 and pH 6.0 by t-test, n.s.—not significant.
